# Brazilian Network for HIV Drug Resistance Surveillance: a survey of individuals recently diagnosed with HIV

**DOI:** 10.1186/1758-2652-12-20

**Published:** 2009-09-18

**Authors:** Lilian A Inocencio, Anderson A Pereira, Maria Cecilia A Sucupira, José Carlos C Fernandez, Célia P Jorge, Denise FC Souza, Helena T Fink, Ricardo S Diaz, Irina M Becker, Theodoro A Suffert, Monica B Arruda, Olinda Macedo, Mariangela BG Simão, Amilcar Tanuri

**Affiliations:** 1 Brazilian DST/AIDS Program, Ministry of Health, Brasilia, DF, Brazil; 2 Lab de Retrovirologia, UNIFESP, Sao Paulo, SP, Brasil; 3 Lab de Imunologia, IOC-FIOCRUZ, RJ, Brazil; 4 Laboratório de Biologia Molecular, UFBA, Salvador, BA, Brazil; 5 Public Health Lab (LACEN) Brasilia, DF, Brazil; 6 Public Health Lab (LACEN), Porto Alegre, RS, Brazil; 7 Health Center Porto, Municipatility of Porto Alegre, Porto Alegre, RS, Brazil; 8 Lab Virologia Molecular, IB-UFRJ, Rio de Janeiro, RJ, Brazil

## Abstract

Use of antiretrovirals is widespread in Brazil, where more than 200,000 individuals are under treatment. Although general prevalence of primary antiretroviral resistance in Brazil is low, systematic sampling in large metropolitan areas has not being performed.

The HIV Threshold Survey methodology (HIV-THS, WHO) was utilized, targeting Brazil's four major regions and selecting the six most populated state capitals: Sao Paulo, Rio de Janeiro, Salvador, Porto Alegre, Brasilia and Belem. We were able to sequence samples from 210 individuals with recent HIV diagnosis, 17 of them (8.1%) carrying HIV isolates with primary antiretroviral resistance mutations. Five, nine and four isolates showed mutations related to resistance to nucleoside reverse transcriptase inhibitors (NRTIs), non-nucleoside reverse transcriptase inhibitors (NNRTIs) and protease inhibitors (PIs), respectively. Using HIV-THS, we could find an intermediate level of transmitted resistance (5% to 15%) in Belem/Brasilia, Sao Paulo and Rio de Janeiro. Lower level of transmitted resistance (<5%) were observed in the other areas. Despite the extensive antiretroviral exposure and high rates of virologic antiretroviral failure in Brazil, the general prevalence of primary resistance is still low. However, an intermediate level of primary resistance was found in the four major Brazilian cities, confirming the critical need to start larger sampling surveys to better define the risk factors associated with transmission of resistant HIV.

## Findings

In the mid '90s, the Brazilian government took a major step in the fight against HIV/AIDS, making antiretrovirals available free to all infected individuals. In addition, a strong prevention programme was established to curb new infections. As a result of these strategies, AIDS-related mortality rates, which peaked in 1995/6, have declined continually, and the number of infected individuals stabilized at lower figures, which contradicts earlier worst-case predicted scenarios [1].

However, given the sequential use of antiretroviral drugs in many patients at the beginning of this programme, the proportion of patients experiencing virologic failure is assumed to be high. In fact, one study, done in 2002 with patients on treatment in 1999/2000, showed that the median time for benefit of initial treatment was approximately 14 months among treatment-naïve patients [2]. Another study revealed that only 27.5% of patients maintained undetectable viral loads after one year of follow up. With the introduction of new drugs and more potent regimens, the level of efficacy of initial treatment increased substantially in Brazil.

HIV primary and secondary antiretroviral resistance is of major concern in a country such as Brazil, where there is widespread access to antiretroviral therapy. A study conducted in Brazil, which analyzed 2474 samples from patients on highly active antiretroviral treatment and who had virologic failure, showed that 95% presented with mutations related to antiretroviral resistance and of them, 21%, 45% and 34% presented resistance to one, two or three classes of antiretroviral drugs, respectively [3].

In contrast, the frequency of primary resistance is much lower. Several independent surveys carried out in Brazilian cities, which analyzed drug-naïve populations selected from recently and chronically infected individuals, showed resistance rates varying from 1.4% to 8.3% [4,5]. These numbers are not different from that observed in developed countries, where transmission of resistant viruses to one or more antiretroviral agent has been reported since 1993.

For instance, surveys conducted in developed countries during the past decade and targeting recent seroconverters have shown prevalence rates of 10% to 17% in France, 13% in German, 14% in the United Kingdom, 15% to 26% in North America, and 23% to 26% in Spain [6-17].

Recent surveys performed in the USA and Europe in comparable populations pointed out a slight increase in primary resistance prevalence, especially to non-nucleoside reverse transcriptase inhibitors (NNRTIs) [10]. A contrast to this picture is the low prevalence of primary drug-resistance mutation in South America and the Caribbean (<4%) [18-20].

In order to monitor the transmission of drug-resistant strains and the subtype profile in Brazil, a National Network for Drug Resistance Surveillance (HIV-BResNet) in the chronically infected drug-naïve population was established in 2000. The first survey, done in 2001, showed an overall primary resistance rate of 6.6% with even distribution of NRTI, NNRTI and protease inhibitor (PI) resistance-related mutations [21]. Here, we describe the results of a new HIV-BResNet survey, conducted in 2007/8 on recently diagnosed HIV patients seeking treatment in AIDS clinics located in six major cities.

Threshold Survey developed by the World Health Organization (WHO), was utilized to compare primary resistance levels in patients in these cities. In this study, we targeted the four major country regions by selecting the six most populated state capitals (Sao Paulo, Rio de Janeiro, Salvador, Porto Alegre, Brasilia and Belem), accounting for more than 70% of the Brazilian HIV/AIDS epidemic and the majority of patients under antiretroviral treatment. The major clinical labs, where most recent diagnosed individuals have their first CD4+ T cell counts and viral loads determinations, were selected to conduct the patient selection, as well as genotypic analysis.

The inclusion criteria was homogeneous across the sites: participating individuals had to have received their HIV diagnosis in the past six months, not have been exposed to antiretroviral drugs for any reason, and had to be more than 18 years old. Individuals were selected to the study and were invited to read and sign informed consents. Samples were collected sequentially until 47 samples were reached and the questionnaire was completed. Samples were analyzed in the same order of inclusion in each site and were amplified and sequenced. This study received an institutional review board (IRB) approval (CONEP, IRB#9772), and informed consents were obtained from all participating individuals.

For genotypic analysis ViroSeq™ v2.0 (Celera Diagnostics, Alameda, California, USA) or TrueGene™ (Siemens Diagnostics, USA) were used. Mutation lists, as well as FASTA files generated by commercial software, were further evaluated according to published guidelines, which exclude common polymorphisms [22]. For subtype assignment, we first separated protease and reverse transcriptase coding regions and submitted the data to the Stanford HIVDR website. For quality assurance purposes, we re-sequenced 10% of samples in target sites..

The sample size and statistical approach from this survey was adapted from WHO's HIV drug resistance Threshold Survey (HIV DR THS) protocol [23]. Each city or region was treated as an independent sampling event, and a binomial sequential strategy was utilized: 47 consecutive HIV-positive specimens were collected and genotyped. The HIV drug resistance prevalence can be categorized into three different levels: low (<5%), moderate (5% to 10%), and high (>15%). The analysis can be stopped at the 34 initial specimens if the prevalence can be categorized as low (<5%, if n ≤ 1) or high (>15%, if n ≥ 6). If it is not met, an additional 13 specimens need to be genotyped to precisely calculate the prevalence categories.

We were able to study 223 individuals in six sites. Their clinical and demographic characteristics are depicted in Table 1. The male:female ratio of our selected individuals was 0.95:1.05, and the average age was 36 years old. The average CD4+ T cell count was 577 cells/mm^3^, which suggests that the selected individuals were in the initial, asymptomatic stage of HIV infection.

**Table 1:** Epidemiological and subtype distribution of individuals included in this survey

**Epidemiological data **		**Subtype distribution (%)**					
	
**Characteristics**	**Number **		**B**	**C**	**F**	**URF**	**Others**
	
Gender (male)	45%	Sao Paulo (n = 47)	82	6	6	6	0
	
Age (years)	36 ± 8	Rio de Janeiro (n = 47)	87	2	2	9	0
	
**Risk factor for HIV (%)**		Salvador (n = 34)	70	3	21	3	3
	
Homosexual	23.6	Porto Alegre (n = 34)	28	69	0	3	0
	
Bisexual	11	Belem/Brasilia (n = 48)	84	6	6	4	0
	
Heterosexual	56.1						
						
Intravenous drug user	0						
						
Blood transfusion	1.6						
						
Other/multiple	8.7						
						
**Biological parameters**							
						
CD4 (cells/mm^3^)^a^	575 ± 221						
						
Viral load (log of copies/ml)^b^	4.58 ± 4.75						

^a, and b ^Calculation based on 187 and 156 individuals who responded to this item on the questionnaire, respectively.

We could successfully amplify and sequence 210 specimens, and found 17 (8.1%) isolates carrying primary drug-resistance mutations. Five, nine and four isolates showed mutations related to resistance to NRTIs, NNRTIs and PIs, respectively. The more prevalent were mutations related to NNRTI (K103N and Y188L/I), followed by NRTI and PI (Table 2). The main PI mutation found was M46I (three individuals), followed by L90M (one individual). Interestingly, most PI mutations were found in Rio de Janeiro. There was a clear geographic trend in primary resistance. We found an intermediate level of transmitted resistance (5% to 15%) in Belem/Brasilia, Sao Paulo and Rio de Janeiro, contrasting with the other sites, where a lower level of transmitted resistance (<5%) was observed. Comparing this prevalence with the data obtained in the BResNet survey performed in 2002, it can be observed that the primary resistance in Brazil remains at a stable level, 6.6% in 2002 versus 8.1 in this latest BResNet survey. In support of this fact, lower levels of transmitted resistance have been detected in other surveys done in major cities, such as Sao Paulo, Rio de Janeiro and Porto Alegre [24-26]. On the other hand, other studies have demonstrated distinct levels of transmitted resistance in other geographic areas; for example, higher levels of primary resistance have been detected in Santos and Salvador [27,28].

**Table 2:** Genotypic distribution of primary mutations and level of resistance per site

Site	Number of primary mutation found			
	**NRTI**	**NNRTI**	**PI**	**Level of Resistance**^1^
		
Sao Paulo (n = 3)				5%-15%

SP015		K103N	M46I	
SP022		K103N, K238T		
SP041		K103N		
Rio de Janeiro (n = 7)				5%-15%
RJ406		K103N		
RJ529			L90M	
RJ565	T69D			
RJ578	T215E, K219R			
RJ026			M46I	
RJ064	M41L, T215C/S			
RJ067			M46I	
Brasilia/Belem (n = 3)				5%-15%

BE010		K103N		
DF 058	M184V/M			
Salvador (n = 1)				< 5%

SA023		K103N		
Porto Alegre (n = 1)				< 5%

PA020			V82A	

This study was not designed to evaluate the subtype distribution and the sample size was very limited in each of the sites sampled. However, we were able to observe that the subtype distribution found closely reflects previous data generated in Brazil. Subtype B was the more prevalent subtype found in all cities sampled (72%), except for Porto Alegre, where a large proportion of HIV-1 subtype C was observed (69%). This rate seems higher than the rate described in this city in HIV ResNet in 2002 (45%), suggesting an increase of this non-B subtype in this country region [21].

Subtype F and several BF mosaics as well Unique Recombinant Forms (URFs) are the other major non-B strain spread throughout the country and corresponds to approximately 13% of the samples analyzed in our study. Interestingly, a CRF2 (A/G) isolate was found in Salvador Bahia, corroborating previous reports regarding the introduction of this west African strain in Brazil [29].

Some studies have demonstrated that the time to obtain a viral load below the detection limit may be increased among individuals with primary resistance [30]. Regardless of high antiretroviral exposure levels and high rates of virologic antiretroviral failure found in Brazil, the general prevalence of primary antiretroviral resistance is still low. However, intermediate level of primary resistance was found in our four major cities, suggesting that more noticeable resistance is transmitted in these areas.

Of note, mutation K103N had a significant increase when the rate observed in 2002 (0.24%) is compared to the one found this study (3.3%; P < 0.001 chi-square). This fact probably reflects the widespread use of efavirenz in the first drug regimen in Brazil. Therefore, it is critical to start larger sampling surveys to better define the risk factors associated with transmission of primary resistance, as well as strengthen the prevention programmes targeting HIV-positive individuals in antiretroviral therapy. An increased prevalence of K103N can be a risk in the long run for the usage of NNRTI-based first-line antiretroviral regimens in Brazil.

## Competing interests

The authors declare that they have no competing interests.

## Authors' contributions

MCS, JCF, CPJ, HF, IMB, TAS, MBA and OM carried out the molecular genetic studies in the six states on BResNet. AAP and DFCS participated in the sequence alignment. AAP participated in the sequence alignment and subtype analysis. RSD and AT participated in the design of the study and performed the statistical analysis. LAI and MBGS conceived of the study, participated in its design and coordination, and helped to draft the manuscript. All other authors in the HIV-BResNet Working Group are professionals involved in the clinical and laboratories sites; they participated in the coordination of this study at site level. All authors read and approved the final manuscript.

## References

[B1] Teixeira PR Vitória MA Barcarolo J Antiretroviral treatment in resource-poor settings: the Brazilian experience AIDS 2004 12 Suppl 3 5 7 10.1097/00002030-200406003-00002 15322477

[B2] Medeiros R Diaz RS Castelo A Estimating the length of the first antiretroviral therapy regiment durability in Sao Paulo, Brazil Braz J Infect Dis 2002 12 298 304 12585973 10.1590/s1413-86702002000600005

[B3] Caseiro MM Golegã AAC Etzel A Diaz RS Characterization of virologic failure after an initially successful 48-week course of antiretroviral therapy in HIV/AIDS outpatients treated in Santos, Brazil Brazilian Journal of Infectious Diseases 2008 12 162 166 10.1590/s1413-86702008000300001 18833397

[B4] De Medeiros LB Lacerda HR Cavalcanti AM De Albuquerque M de-F Primary resistance of human immunodeficiency virus type 1 in a reference center in Recife, Pernambuco, Brazil Mem Inst Oswaldo Cruz 2006 12 845 849 17293977 10.1590/s0074-02762006000800004

[B5] Brígido LF Nunes CC Oliveira CM Knoll RK Ferreira JL Freitas CA Alves MA Dias C Rodrigues R Research Capacity Program HIV type 1 subtype C and CB Pol recombinants prevail at the cities with the highest AIDS prevalence rate in Brazil AIDS Res Hum Retroviruses 2007 12 1579 1586 10.1089/aid.2007.0102 18160017

[B6] Chaix ML Harzic M Masquelier B Pellegrin I Meyer L Costagliola DI Rouzioux C Brun-Vezinet F Ac11 Resistance Group Cohort, Primo and Primoferon Study Groups Prevalence of genotypic drug resistance among French patients infected during the year 1999 Eighth Conference on Retroviruses and Opportunistic Infections. Chicago 2001 2 4

[B7] Tamalet C Pasquier C Yahi N Colson P Martin LP Lepeu G Quinson AM Poizot-Martin I Dhiver C Fantini J Prevalence of drug resistant mutants and virological response to combination therapy in patients with primary HIV-1 infection J Med Virol 2000 12 181 186 10.1002/(sici)1096-9071(200006)61:2<181::aid-jmv2>3.0.co;2-t 10797372

[B8] Duwe S Brunn M Altman D Hamouda O Schmidt B Walter H Pauli G Kücherer C Frequency of genotypic and phenotypic drug-resistant HIV-1 among therapy-naive patients from the German seroconverter study J Acquir Immune Defic Syndr 2001 12 266 273 10.1097/00042560-200103010-00010 11242200

[B9] UK Collaborative Group on Monitoring the Transmission of HIV Drug Resistance Analysis of prevalence of HIV-1 drug resistance in primary infections in the United Kingdom Br Med J 2001 12 1087 1088 10.1136/bmj.322.7294.1087 PMC31258 11337435

[B10] Little SJ Routy JP Daar ES Markowitz M Collier AC Koup RA Conway B Saag MS Connick E Holte S Corey L Keiser PH Mwatha A Dawson K Whitcomb JM Hellmann NS Richman DD Antiretroviral drug susceptibility and response to initial therapy among recently HIV-infected subjects in North America Antiviral Ther 2001 12 Suppl 1 21

[B11] Boden D Hurley A Zhang L Cao Y Guo Y Jones E Tsay J Ip J Farthing C Limoli K Parkin N Markowitz M HIV-1 drug resistance in newly infected individuals JAMA 1999 12 1135 1141 10.1001/jama.282.12.1135 10501116

[B12] Brodine SK Shaffer RA Starkey MJ Tasker SA Gilcrest JL Louder MK Barile A VanCott TC Vahey MT McCutchan FE Birx DL Richman DD Mascola JR Drug resistance patterns, genetic subtypes, clinical features, and risk factors in military personnel with HIV-1 seroconversion Ann Intern Med 1999 12 502 506 10507958 10.7326/0003-4819-131-7-199910050-00004

[B13] Briones C Perez-Olmeda M Rodriguez C Romero J Hertogs K Soriano V Primary genotypic and phenotypic HIV-1 drug resistance in recent seroconverters in Madrid J Acquir Immune Defic Syndr 2001 12 145 150 10.1097/00042560-200102010-00006 11242181

[B14] Weinstock H Respess R Heneine W Petropoulos CJ Hellmann S Luo CC Pau CP Woods T Gwinn M Kaplan J Prevalence of mutations associated with reduced antiretroviral drug susceptibility among human immunodeficiency virus type 1 seroconversters in the United States, 1993-1998 J Infect Dis 2000 12 330 333 10.1086/315686 10882618

[B15] Zaidi I Weinstock H Woods T Petropoulos CJ Hellmann NS Luo CC Pau CP Woods T Gwinn M Kaplan J Prevalence of mutations associated with antiretroviral drug resistance among HIV-1-infected persons in 10 US cities, 1997-2000 Antiviral Ther 2001 12 Suppl 1 118

[B16] Puig T Perez-Olmeda M Rubio A Ruiz L Briones C Franco JM Gómez-Cano M Stuyver L Zamora L Alvarez C Leal M Clotet B Soriano V Prevalence of genotypic resistance to nucleoside analogues and protease inhibitors in Spain AIDS 2000 12 727 732 10.1097/00002030-200004140-00012 10807196

[B17] Gomez-Cano M Rubio A Puig T Perez-Olmeda M Ruiz L Soriano V Pineda JA Zamora L Xaus N Clotet B Leal M Prevalence of genotypic resistance to nucleoside analogues in antiretroviral-naive and antiretroviral-experienced HIV-infected patients in Spain AIDS 1998 12 1015 102010.1097/00002030-199809000-00007 9662197

[B18] Kijak GH Pampuro SE Avila MM Zala C Cahn P Wainberg MA Salomon H Resistance profiles to antiretroviral drugs in HIV-1 drug-naive patients in Argentina Antiviral Ther 2001 12 71 77 11417764

[B19] Cesaire R Dos Santos G Abel S Bera O Sobesky G Cabie A Drug resistance mutations among HIV-1 strains from antiretroviral-naive patients in Martinique, French West Indies J Acquire Immune Defic Syndr 1999 12 401 405 10.1097/00126334-199912010-00012 10634203

[B20] Delgado E Leon-Ponte M Villahermosa ML Cuevas MT Deibis L Echeverria G Thomson MM Pérez-Alvarez L Osmanov S Nájera R Analysis of HIV type 1 protease and reverse transcriptase sequences from Venezuela for drug resistance-associated mutations and subtype classification: A UNAIDS study AIDS Res Hum Retroviruses 2001 12 753 758 10.1089/088922201750237022 11429115

[B21] Brindeiro RM Diaz RS Sabino EC Morgado MG Pires IL Brigido L Dantas MC Barreira D Teixeira PR Tanuri A Brazilian Network for Drug Resistance Surveillance Brazilian Network for HIV Drug Resistance Surveillance (HIV-BResNet): a survey of chronically infected individuals AIDS 2003 12 1063 1069 10.1097/00002030-200305020-00016 12700457

[B22] Bennett DE Camacho RJ Otelea D Kuritzkes DR Fleury H Kiuchi M Heneine W Kantor R Jordan MR Schapiro JM Vandamme AM Sandstrom P Boucher CA Vijver D van de Rhee SY Liu TF Pillay D Shafer RW Drug resistance mutations for surveillance of transmitted HIV-1 drug-resistance: 2009 update PLoS ONE 2009 12 e4724 10.1371/journal.pone.0004724 19266092 PMC2648874

[B23] Myatt M Bennett DE A novel sequential sampling technique for the surveillance of transmitted HIV drug resistance by cross-sectional survey for use in low resource settings Antivir Ther 2008 12 Suppl 2 37 48 18575190

[B24] Gonsalez CR Alcalde R Nishiya A Barreto CC Silva FE de Almeida A Mendonça M Ferreira F Fernandes SS Casseb J Duarte AJ Drug resistance among chronic HIV-1-infected patients naïve for use of anti-retroviral therapy in Sao Paulo city Virus Res 2007 12 87 90 10.1016/j.virusres.2007.06.021 17686543

[B25] Pires IL Soares MA Speranza FA Ishii SK Vieira MC Gouvêa MI Guimarães MA de Oliveira FE Magnanini MM Brindeiro RM Tanuri A Prevalence of human immunodeficiency virus drug resistance mutations and subtypes in drug-naive, infected individuals in the army health service of Rio de Janeiro, Brazil J Clin Microbiol 2004 12 426 430 10.1128/JCM.42.1.426-430.2004 14715797 PMC321664

[B26] Rodrigues R Scherer LC Oliveira CM Franco HM Sperhacke RD Ferreira JL Castro SM Stella IM Brigido LF Low prevalence of primary antiretroviral resistance mutations and predominance of HIV-1 clade C at polymerase gene in newly diagnosed individuals from south Brazil Virus Res 2006 12 201 217 10.1016/j.virusres.2005.10.004 16332398

[B27] Sucupira MC Caseiro MM Alves K Tescarollo G Janini LM Sabino EC Castelo A Page-Shafer K Diaz RS High levels of primary antiretroviral resistance genotypic mutations and B/F recombinants in Santos, Brazil AIDS Patient Care STDS 2007 12 116 128 10.1089/apc.2006.0079 17328661

[B28] Pedroso C Queiroz AT Alcântara LC Drexler JF Diaz RS Weyll N Brites C High prevalence of primary antiretroviral resistance among HIV-1-infected adults and children in Bahia, a northeast state of Brazil J Acquir Immune Defic Syndr 2007 12 251 253 10.1097/QAI.0b013e318050d8b0 17527096

[B29] Eyer-Silva WA Morgado MG Autochthonous horizontal transmission of a CRF02_AG strain revealed by a human immunodeficiency virus type 1 diversity survey in a small city in inner state of Rio de Janeiro, Southeast Brazil Mem Inst Oswaldo Cruz 2007 12 809 815 17992366 10.1590/s0074-02762007005000112

[B30] Little SJ Smith DM HIV treatment decisions and transmitted drug resistance Clin Infect Dis 2005 12 233 235 10.1086/431215 15983921

